# Dietary Phytonutrients in Fibromyalgia: Integrating Mechanisms, Biomarkers, and Clinical Evidence—A Narrative Review

**DOI:** 10.3390/medicina61122211

**Published:** 2025-12-15

**Authors:** Tuba Kahraman, Aylin Ayaz

**Affiliations:** 1 Department of Nutrition and Dietetics, Faculty of Health Sciences, Halic University, İstanbul 34060, Türkiye; 2 Department of Nutrition and Dietetics, Faculty of Health Sciences, Hacettepe University, Ankara 06800, Türkiye; aylinayazbdb@gmail.com

**Keywords:** fibromyalgia, nutrition, phytonutrient, oxidative stress, inflammation, SIRT1, gut microbiota

## Abstract

*Background and Objectives*: Fibromyalgia (FM) is associated with chronic pain, oxidative stress, low-grade inflammation, and disturbances in signalling along the gut–brain axis. These pathways may be modulated by plant-derived phytonutrients. This narrative review summarises mechanistic and clinical evidence on phytonutrient-based strategies in FM. *Materials and Methods*: Following SANRA guidelines, we searched PubMed, Web of Science, Scopus and ScienceDirect for human and relevant preclinical studies published between 2005 and October 2025 that evaluated phytonutrient-rich dietary patterns or isolated bioactives in relation to FM symptoms or underlying mechanisms. *Results*: There is a consistent association between FM and increased oxidative damage and reduced antioxidant defences. Adopting plant-based diets, particularly Mediterranean-type and low-FODMAP diets, has been linked to improvements in pain, fatigue, sleep, and gastrointestinal symptoms, as well as modest gains in quality of life. However, the effects on inflammatory markers are conflicting. Trials of selected bioactive compounds, such as coenzyme Q10, curcumin-based formulations, L-carnitine and certain probiotics, suggest beneficial effects on symptoms, whereas others show little or no effect. Studies of the microbiome indicate a loss of butyrate-producing bacteria and altered microbial metabolites. Early dietary or probiotic interventions may partially mitigate these changes to some extent. Preclinical studies have identified SIRT1 as a potential mediator, but there is a lack of human data. Reporting on safety, dosage and formulation is often inadequate. *Conclusions*: Given the narrative design of this review and the methodological heterogeneity of the included studies, the overall certainty of the evidence cannot be formally graded and should be regarded as limited and heterogeneous. Nevertheless, current data supports phytonutrient-rich, food-based approaches as adjuncts rather than alternatives to standard FM care. Well-designed randomised trials with standardised outcomes and reporting of dose, formulation and relevant biomarkers are needed to identify the most effective strategies and the patient subgroups most likely to benefit.

## 1. Introduction

Fibromyalgia (FM) is a chronic, multifactorial syndrome characterised by widespread musculoskeletal pain, fatigue, sleep disturbances, gastrointestinal symptoms, cognitive impairment, and symptoms of anxiety and depression [1]. An estimated 2.10% of the global population is affected by FM, which is more prevalent in women [2]. The development of the condition is thought to be influenced by central nervous system sensitisation, neuroinflammation, hormonal imbalances, oxidative stress and disturbances in the gut microbiota [1,3,4]. Recent studies have shown that gut dysbiosis may increase intestinal permeability and reduce the production of short-chain fatty acids (SCFAs), thereby promoting inflammation and heightened pain sensitivity [4,5,6].

At the molecular level, sirtuin-1 (SIRT1), a protein dependent on nicotinamide adenine dinucleotide (NAD^+^) with key roles in the cellular stress response and energy metabolism, has emerged as a potential regulator in the pathophysiology of FM [7]. SIRT1 contributes to the maintenance of cellular homeostasis through its anti-inflammatory and antioxidant actions [8]. Furthermore, in individuals with chronic pain, both serum and spinal cord SIRT1 levels have been reported to be inversely correlated with pain intensity ratings [9,10,11].

Phytonutrients, also referred to as phytochemicals, are secondary plant metabolites that are produced in response to environmental stressors and other environmental changes [12]. Key dietary sources include fruit, vegetables, legumes, nuts, tea and coffee [13]. Numerous epidemiological studies have shown that a high intake of plant-based foods is associated with a lower risk of chronic disease and mortality [14,15,16]. In this context, phytonutrients, which are notable for their antioxidant, anti-inflammatory, and neuromodulator properties [17], have been proposed as potential agents for alleviating FM symptoms [7]. Compounds such as polyphenols, flavonoids, carotenoids, and glucosinolates may target mechanisms involved in the pathophysiology of FM by supporting SIRT1 activation [18] and modulating gut microbiota composition [19]. Despite growing interest, the evidence base is fragmented: human studies are generally small and heterogeneous, whereas data from animal models are more robust but have limited clinical translatability [20]. Against this background, the present narrative review aims to: (i) synthesise mechanistic and preclinical data on how phytonutrients modulate oxidative stress, inflammation, gut microbiota and SIRT1-related pathways relevant to FM; (ii) summarise and critically appraise clinical evidence from dietary pattern and isolated phytonutrient interventions on FM symptoms and biomarkers; and (iii) identify key gaps to guide future biomarker-anchored trials and clarify the potential role of phytonutrient-rich dietary strategies as adjuncts to standard FM care.

## 2. Materials and Methods

### 2.1. Literature Search and Study Selection

This narrative review was conducted in accordance with the SANRA (Scale for the Assessment of Narrative Review Articles) guidelines [21]. A comprehensive literature search was undertaken in PubMed (National Center for Biotechnology Information, Bethesda, MD, USA), Web of Science (Clarivate Analytics, Philadelphia, PA, USA), Scopus (Elsevier, Amsterdam, The Netherlands) and ScienceDirect (Elsevier, Amsterdam, The Netherlands) to identify articles published between 1 January 2005 and 16 October 2025. Only studies with full-text available in English or Turkish were considered. Google Scholar (Google LLC, Mountain View, CA, USA) was used solely for forward citation tracking; for each key paper, approximately the first 200 potentially relevant records were screened. Grey literature (e.g., theses, reports) and preprints were not included. Eligible publications investigated the potential associations between phytonutrients—including polyphenols, carotenoids, glucosinolates, and organo-sulphur compounds—and FM through mechanisms such as oxidative stress, inflammation, modulation of the gut microbiota, or SIRT1 regulation, in human studies or animal models. In vitro studies, editorials, reviews without primary data, studies without accessible full texts, and studies whose source language was neither English nor Turkish, were excluded (Table 1).

All records were deduplicated using Mendeley Cite (version 1.69.1; Elsevier Ltd., London, UK) and screened by the authors based on title, abstracts and full-text content to ensure methodological relevance and quality. The evidence was organised according to study model and mechanistic pathway, with particular attention to consistency, limitations and translational implications. Supplementary File collates the supporting materials. Table S1 details the Boolean search strategies and keywords; Table S1B describes the data charting form (extraction fields); Section S1C outlines the deduplication rules; Section S1D lists the predefined reasons for exclusion. In addition, the reference lists of the included studies were manually searched to identify further relevant research, including randomised controlled trials, systematic reviews, meta-analyses, prospective cohorts and experimental studies.

### 2.2. Study Quality and Risk of Bias Appraisal

Due to the scope of this narrative review and the heterogeneity of the methodologies used in the included studies, we did not apply a single quantitative risk-of-bias tool (e.g., the Cochrane instruments). Instead, we adopted a pragmatic two-step quality assessment approach. First, we restricted eligibility to primary studies published in peer-reviewed journals indexed in major international databases (e.g., Web of Science Core Collection and/or Scopus) and ranked within the top three quartiles (Q1–Q3) of their subject category according to the most recent Journal Citation Reports (Clarivate Analytics, Philadelphia, PA, USA) or Scimago Journal Rank (Scimago Lab, Madrid, Spain) at the time of the search. Articles from non-indexed or potentially questionable journals were excluded. Secondly, within this pool, we qualitatively assessed the following methodological features: clarity of FM diagnosis; sample size; description and plausibility of phytonutrient exposure (dietary pattern or bioactive compound); appropriation of comparators; outcome measures (symptoms and biomarkers); and basic control of confounding factors. Major methodological limitations and potential sources of bias are reported in the Limitations.

## 3. The Pathophysiology of Fibromyalgia: An Overview

Fibromyalgia is a chronic syndrome with a multifactorial pathophysiological basis, the aetiology of which is not yet fully understood [1]. Current evidence suggests that FM is closely related to disturbances in pain processing within the central nervous system (CNS). Patients typically exhibit heightened sensitivity to nociceptive stimuli in the CNS, which has been liked to elevated levels of excitatory neurotransmitters, such as glutamate and substance P, and reduced levels of inhibitory neurotransmitters, including serotonin and noradrenaline. In addition, abnormalities in central pain-modulatory pathways, such as dopamine dysregulation and functional impairments in the endogenous opioid system, have been reported [22].

Abnormalities in muscle, connective tissue and visceral structures at the peripheral level can trigger central sensitization by increasing nociceptive tonic support in the spinal cord. Other important factors in the development of the condition include neuroendocrine imbalances, genetic predisposition, environmental triggers and psychosocial stressors [23,24]. Recent studies indicate that oxidative stress [3,25] and inflammation [26,27] may also contribute to the pathogenesis of FM. Elevated levels of reactive oxygen species (ROS) in patients with FM may impair mitochondrial function and energy metabolism in muscle and nerve cells, thereby contributing to chronic, widespread pain [28], while markers of lipid peroxidation have been positively correlated with FM severity scores [29,30]. Furthermore, pro-inflammatory cytokines have been reported to promote nociceptive activation in FM. In particular, increased concentrations of the pro-inflammatory cytokines tumour necrosis factor-alpha (TNF-α), interleukin-6 (IL-6), and interleukin-8 (IL-8), as well as alterations in the anti-inflammatory cytokine interleukin-10 (IL-10) and specific chemokines, have been described [27]. These intertwined pathways, oxidative stress, low-grade inflammation and neuroimmune-driven sensitisation represent biologically plausible targets for modulation by phytonutrient-rich dietary patterns and specific bioactive compounds [3,17,18,19,25,26,27,28,29,30].

Disruptions to the gut microbiota are believed to play a role in the development of FM by affecting the gut–brain axis [31,32]. Compared with healthy individuals, people with FM display a characteristic dysbiosis, including a reduced abundance of taxa that produce SCFAs and support the intestinal barrier, such as *Faecalibacterium prausnitzii*, *Lachnospiraceae*, *Ruminococcaceae* and *Bifidobacteriaceae* together with an increased abundance of *Clostridia* species. Alterations in microbial metabolites, including SCFAs and bile acids, have also been reported [4,33,34]. These compositional and metabolic changes have been linked to greater pain intensity and fatigue, suggesting that gut microbiota alterations are associated with the severity of FM symptoms and may contribute to its pathophysiology via the gut–brain axis [33,34,35]. 

## 4. Effects of Phytonutrients on Mechanisms Related to FM

Phytonutrients are secondary plant metabolites that belong to diverse phytochemical classes, such as polyphenols, terpenoids, carotenoids, glucosinolates and organosulfur compounds [36,37,38]. Although they are not essential nutrients in the classical sense, higher intakes of phytonutrient-rich plant foods are consistently associated with a lower risk of chronic diseases [38].

Many of these compounds exert pleiotropic biological effects that are directly relevant to FM, including indirect antioxidant activity, modulation of inflammatory and immune pathways, effects on mitochondrial function and cellular stress responses, interactions with SIRT1 signalling and bidirectional crosstalk with the gut microbiota [37,38,39,40,41,42]. Different colour groups of vegetables and fruits tend to be enriched in distinct phytonutrient profiles (e.g., anthocyanin-rich red–purple berries, carotenoid-rich orange–yellow produce, glucosinolate-rich green cruciferous vegetables), which together provide complementary antioxidant, anti-inflammatory and neuromodulatory activities [39,43,44,45,46,47,48,49,50,51,52,53,54]. Thus, a varied, “colourful” plant-based dietary pattern may represent a practical food-based strategy to increase phytonutrient exposure and to target the key mechanisms implicated in FM pathophysiology.

**Figure 1:**
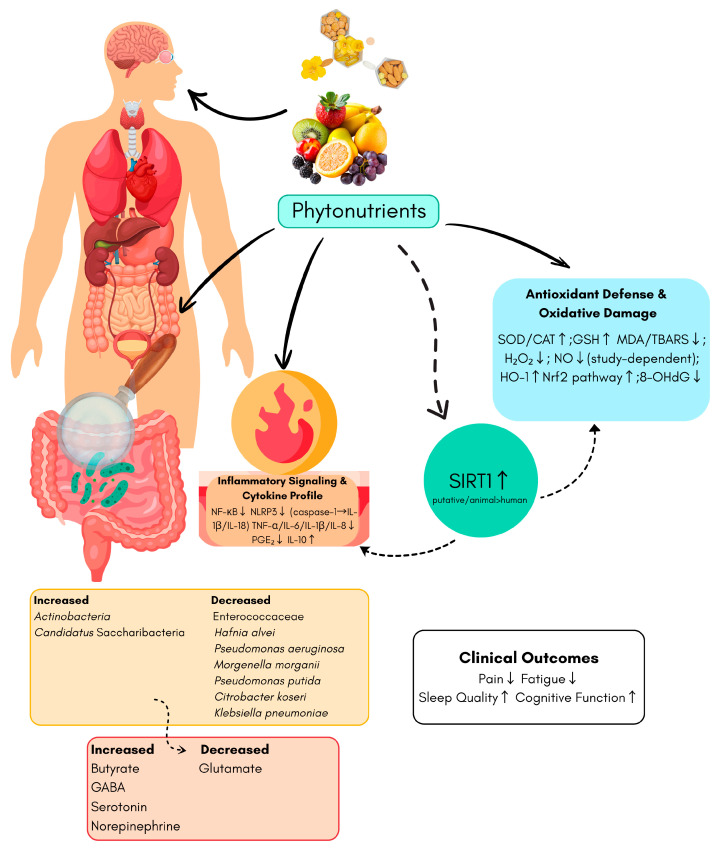
The Role of Phytonutrients in Modulating Fibromyalgia-Related Pathophysiological Mechanisms. This is an original illustration created by the authors (using Canva (Canva Pty Ltd., Sydney, Australia)), based on mechanisms described in the cited studies. The phytonutrients in the centre (polyphenols, carotenoids, glucosinolates, organo-sulphur compounds) may activate SIRT1 and related pathways to reduce oxidative stress (Nrf2 ↑; antioxidant molecules SOD/CAT/GSH ↑; lipid peroxidation: MDA/TBARS ↓; nitrosative damage: 8-OHdG ↓) and inflammation (NF-κB/NLRP3 ↓; TNF-α/IL-6/IL-1β/PGE_2_ ↓; IL-10 ↑). In the microbiota–metabolite axis, SCFAs (especially butyrate) ↑, GABA/glutamate balance and serotonin levels may be modulated favourably. These biological effects are associated with reduced pain and fatigue, and improved sleep and cognitive function. Antioxidant panel lists systemic markers reported in included studies; subcellular localization is not implied. Dashed arrows indicate putative/limited human evidence; ↑/↓ symbols indicate the reported direction of effect. Abbreviations: SIRT1: Sirtuin-1; Nrf2: Nuclear factor erythroid 2–related factor 2; NF-κB: Nuclear factor kappa B; NLRP3: NOD-like receptor family pyrin domain containing 3; SCFA: Short-chain fatty acid; SOD: Superoxide dismutase; CAT: Catalase; GSH: Glutathione; MDA: Malondialdehyde; TBARS: Thiobarbituric acid-reactive substances; NO: Nitric oxide; 8-OHdG: 8-hydroxy-2′-deoxyguanosine; PGE_2_: Prostaglandin E_2_; GABA: Gamma-aminobutyric acid.

Fibromyalgia is now widely recognised as a disorder affecting multiple systems in the body. It is characterised by a combination of factors, including redox imbalance, immune activation and inflammasome signalling, microbiota–gut–brain interactions, and SIRT1-mediated metabolic control [1,4,7]. These factors converge on nociceptive sensitisation. Within this framework, phytonutrients may exert beneficial effects through multiple ways, including antioxidant, anti-inflammatory, microbiota-modulating and SIRT1-activating properties (see Figure 1). The subsections below outline the potential therapeutic roles of phytonutrients along these mechanistic axes.

### 4.1. Antioxidant Effects

One of the key mechanisms underlying the pathophysiology of FM is an imbalance between the formation of ROS and the activity of the antioxidant defence system [55]. Total antioxidant capacity (TAC) levels and levels of antioxidant enzymes, such as superoxide dismutase (SOD), catalase (CAT), and glutathione peroxidase (GPx) have been reported to be significantly lower in patients with FM, and these reductions are inversely correlated with disease severity indices, including Fibromyalgia Impact Questionnaire scores, pain ratings and anxiety levels [30,56,57]. This imbalance exacerbates oxidative stress, mitochondrial dysfunction, inflammation and pain sensitivity.

Recent experimental evidence further supports this concept. In an intermittent cold-stress mouse model of FM, Martins et al. (2025) showed that pharmacological modulation of the Nrf2–NF-κB axis with 4-amino-3-(phenylselanyl)benzenesulfonamide (4-APSB) restored redox homeostasis, normalised lipid peroxidation, re-established Na^+^/K^+^-ATPase activity in the central nervous system and attenuated pain-like behaviour, suggesting that impaired Nrf2-driven cytoprotective signalling may contribute to oxidative damage and neuronal dysfunction in FM [58].

Further evidence from human observational studies points to redox disruption, while highlighting considerable heterogeneity. In a case–control study including 30 women with FM and 30 age-matched controls, fasting plasma vitamins and oxidative markers were compared. Clinical scores (tender point number, pain, depression, anxiety and FIQR) were markedly higher in the FM group, while LP levels were significantly elevated and vitamins A and E were significantly lower in patients; vitamin C, β-carotene and NO did not differ between groups. However, these findings are based on single time-point comparisons in a small sample, and the analyses were not adjusted for potential confounders such as BMI, dietary intake or medication use. Therefore, the observed differences in LP and fat-soluble antioxidant vitamins should be interpreted as exploratory associations rather than evidence of a causal role in FM pathophysiology [59]. In another case–control study conducted in Bangladesh, serum malondialdehyde (MDA) and C-reactive protein (CRP) levels were significantly higher in FM than in healthy individuals, whereas vitamin C, calcium, magnesium, zinc and copper levels were lower. These results suggest that FM may be associated with increased oxidative stress and an imbalance of micronutrients and minerals [60]. A study conducted in Turkish patients reported that oxidative stress parameters, namely TAC, total oxidant capacity (TOC), MDA and oxidative stress index (OSI) levels, were significantly higher in FM patients than in healthy controls. However, no relationship was observed between these parameters and disease symptom severity, anxiety, depression or quality of life (QoL), Fibromyalgia Impact Questionnaire (FIQ) scores. On this basis, it appears that, while oxidative stress may be diagnostically informative, they are insufficient to explain the severity of clinical symptoms on their own [61]. Conversely, in another sample of Turkish patients, investigators observed elevated MDA and low-density lipoprotein cholesterol (LDL-C) levels, together with significantly lower Paraoxonase-1 (PON-1) activity, NO and high-density lipoprotein cholesterol (HDL-C) levels. PON-1 activity was negatively correlated with MDA, LDL-C, pain intensity (VAS), FIQ score, tender point count, age and depression score, and positively correlated with NO and HDL-C. MDA levels were positively correlated with pain intensity, FIQ score, tender point count and age, and negatively correlated with NO. Taken together, these findings suggest that the balance between oxidants and antioxidants is disturbed in patients with FM and that this imbalance may influence disease symptoms [62]. Low SOD levels and impaired quality of life were found to be associated with increased muscle pain, while high thiobarbituric acid-reactive substances (TBARS) levels, low muscle performance, and impaired quality of life were associated with decreased lean body mass. Overall, the data imply that imbalances in oxidative stress markers and antioxidant capacity may contribute to muscle pain and loss in patients with FM [25].

Evidence indicates that FM is characterised by disrupted redox homeostasis, including increased lipid peroxidation (MDA, 4-HNE), decreased antioxidant capacity (CoQ10, SOD, CAT), and mitochondrial dysfunction. Preliminary preclinical and small clinical studies suggest potential benefits of NRF2 activators, high-dose thiamine, CoQ10, molecular hydrogen, and oxygen–ozone therapy [63]. In a cohort of 76 patients with myalgic encephalomyelitis/chronic fatigue syndrome (ME/CFS), a condition that shares several clinical and mechanistic features with FM (e.g., chronic pain and fatigue, oxidative and nitrosative stress), nutraceuticals with anti-inflammatory and antioxidant properties (e.g., L-carnitine, coenzyme Q10, taurine, lipoic acid, curcumin, quercetin, N-acetylcysteine and zinc) were associated with reductions in IgM-mediated autoimmune responses against oxidative stress-related epitopes (OSEs) and nitrosative adducts (NO-adducts), together with improvements in symptoms [64]. These findings support the concept that oxidative/nitrosative autoimmune responses may be linked to symptom burden in ME/CFS. However, as the study was uncontrolled, involved a multimodal supplement regimen, and did not include patients with FM, any translational implications for FM remain speculative and require confirmation in randomised controlled trials specifically designed for FM. Building on these observations, a pilot study in female patients with FM evaluated the effects of oral supplementation with vitamin E (DL-α-tocopheryl acetate, 150 mg/day) and vitamin C (ascorbic acid, 500 mg/day) for 12 weeks, either alone or combined with exercise. The intervention reduced plasma and erythrocyte lipid peroxidation levels, while increasing plasma vitamins A and E, reduced glutathione (GSH), and GPx activity. Although these changes indicated an enhancement of the antioxidant defense system, no clinical improvement in FM symptoms was observed. Overall, combined vitamin C and E supplementation, particularly when accompanied by exercise, may attenuate oxidative stress in FM patients [65]. In a randomised, placebo-controlled, crossover trial evaluating the efficacy of alpha-lipoic acid (ALA) in the treatment of FM, no significant difference was found between ALA and placebo in terms of the primary endpoint of pain intensity. Secondary analyses likewise showed no statistically significant differences in the evaluated parameters, including quality of life, FIQ scores, sleep and mood. However, subgroup analysis revealed a significant reduction in pain favouring ALA in men, whereas no such effect was observed in women. Side effects were generally mild and did not differ significantly from those observed with the placebo. Overall, these findings suggest that, at the tested dose and duration, ALA is not an effective stand-alone treatment for FM. More broadly, they emphasise a significant limitation of antioxidant-based strategies: addressing redox imbalance with a single agent does not invariably result in clinically significant symptom relief. This underscores the necessity of biomarker-guided, mechanism-based approaches and adequately powered trials when assessing potential antioxidant therapies for FM [66].

Preclinical models more consistently demonstrate benefits of antioxidant and polyphenol-based agents on FM-relevant endpoints. In a reserpine-induced FM mouse model, oral resveratrol combined with rice bran oil reduced pain-like behaviors and cerebrospinal fluid (CSF) reactive species and produced antidepressant-like effects, supporting an antioxidative mechanism that merits clinical evaluation [67]. Isorhamnetin improved pain–depression phenotypes and cognitive function, while increasing glutathione (GSH) and lowering TBARS and pro-inflammatory cytokines (TNF-α and IL-1β), as well as rebalancing neurotransmitters by decreasing glutamate and increasing serotonin and norepinephrine) [68]. Similarly, anthocyanins have been shown to alleviate pain, depressive-like behaviour, and cognitive impairment in a dose-dependent manner. High-dose anthocyanin treatment (200 mg/kg) increased serotonin levels and reduced oxidative stress (H_2_O_2_), elevated the expression of microRNAs (miR-145-5p and miR-451a), and suppressed spinal neurone degeneration, TNF-α, and caspase-3 activation. On this basis, anthocyanins appear to be a promising candidate for FM owing to their antioxidant, anti-inflammatory, and neuroprotective properties [69]. Nanoformulations of lutein and β-carotene restored GSH and reduced MDA, hydrogen peroxide (H_2_O_2_), and NO in reserpine-induced models, supporting the potential of nano-carotenoids as redox-active candidates [70]. In a model of pain–depression comorbidity induced by chronic constrictive injury (CCI) and chronic unpredictable mild stress (CUMS), gallic acid alleviated tissue iron overload and mitochondrial damage by modulating the P2X7-ROS signalling pathway. This, in turn, suppressed spinal microglial ferroptosis and led to behavioural improvement, suggesting that gallic acid has therapeutic potential in oxidative stress-mediated mechanisms [71]. In addition, fisetin, a flavonoid polyphenol, significantly improved allodynia, hyperalgesia, and depressive-like behaviors in a reserpine-induced FM model by restoring biogenic amine levels (5-HT, noradrenaline, and dopamine) and reducing oxidative and nitrosative stress [72]. Collectively, these findings indicate that antioxidant and polyphenol-based compounds may have therapeutic potential in the management of FM through their modulation of oxidative stress, inflammation, and neurotransmitter homeostasis.

A recent systematic review synthesised clinical evidence in FM and reported pain reduction with several agents, including Fibromyalgine^®^ (a combination of vitamin C, acerola, ginger root, and freeze-dried royal jelly), coenzyme Q10 (alone or with pregabalin), ferric carboxymaltose, vitamins C and E, *Nigella sativa*, magnesium plus amitriptyline, L-carnitine and Sun Chlorella™ [73]. However, the studies had different effect sizes, dosing, and populations, and the methodological quality was inconsistent.

In summary, FM appears to involve disrupted redox homeostasis, characterised by elevated lipid peroxidation (e.g., MDA and 4-HNE), reduced antioxidant defences (e.g., CoQ10, SOD and CAT) and mitochondrial dysfunction, with plausible downstream effects on pain and neuroinflammation. Figure 1 schematically summarises these redox disturbances and their proposed modulation by phytonutrients, which involves enhancing Nrf2-mediated antioxidant defence, suppressing NF-κB/NLRP3-driven inflammation, activating SIRT1, and promoting favourable shifts in the gut microbiota–metabolite axis. Table 2 summarises studies investigating the effects of phytonutrients on oxidative stress mechanisms in FM. Due to the current heterogeneity of clinical data, future research should focus on adequately powered, well-controlled randomised controlled trials (RCTs) that: incorporate redox biomarker panels and standardised outcomes (pain, FIQR, function, mood/sleep); and rigorously control key confounders (diet, physical activity, comorbidities, medications and sex). Until such evidence becomes available, antioxidant-based interventions should be regarded as adjunctive components of a multimodal FM management programme, with careful attention to safety and individual responses.

### 4.2. Anti-Inflammatory Effects

Another pathway implicated in the pathogenesis of FM is inflammation. It has been proposed that pro-inflammatory cytokines lower nociceptive thresholds. Fibromyalgia is most consistently linked to elevated levels of TNF-α, IL-6 and IL-8, as well as to alterations in the anti-inflammatory cytokine IL-10 and selected chemokines [27].

The NLRP3 (NOD-like receptor family pyrin domain containing 3) inflammasome is a protein complex that initiates intracellular inflammatory responses and triggers the release of cytokines, such as IL-1β and IL-18. Excessive or dysregulated activation of the NLRP3 inflammasome may exacerbate inflammation and symptoms in chronic pain conditions such as FM [74,75,76]. In a mouse model relevant to myalgic encephalomyelitis/chronic fatigue syndrome rather than FM, combined lipopolysaccharide administration and swim stress activated the diencephalic NLRP3 inflammasome, leading to caspase-1-mediated IL-1β maturation and fatigue-like motor deficits, while lactate and MDA levels remained unchanged. NLRP3 knock-out reduced these effects, suggesting that an IL-1β-dependent NLRP3/caspase-1 neuroimmune pathway is a key driver of fatigue and providing a rationale for targeting this axis therapeutically [77]. Additionally, in a mouse model of FM, astaxanthin (AST) has been shown to reduce mechanical and thermal pain, as well as to alleviate sleep disturbances and depressive-like behaviours, potentially by decreasing NLRP3 expression along this pathway [78].

In a preclinical reserpine-induced pain–depression model in rats, intraperitoneal curcumin (100–300 mg/kg) improved reduced nociceptive thresholds and depressive-like behaviours, normalising alterations in norepinephrine, serotonin, and dopamine levels. Curcumin also modulated substance P, nitrosative stress markers, inflammatory cytokines (TNF-α and IL-1β), NF-κB and caspase-3 in a dose-dependent manner. These findings suggest that curcumin can attenuate neuroinflammation and nitrosative stress in animal models of pain and depression. However, these results are derived from preclinical studies and cannot be directly extrapolated to the therapeutic efficacy of curcumin in humans with FM [79].

The antinociceptive effects of garlic and organo-sulphur compounds are thought to be mediated, at least in part, by their antioxidant and anti-inflammatory properties. These include inhibition of prostaglandin (PG) synthesis and pro-inflammatory cytokine production, reduction of cyclooxygenase-2 (COX-2) activity, and increased levels of anti-inflammatory cytokines [80]. Quercetin also shows clinical potential for use in the management of nociceptive and inflammatory pain, exhibiting effects similar to local anaesthetics and non-steroidal anti-inflammatory drugs (NSAIDs) [81].

In a mouse model of FM induced by intermittent cold stress, the administration of dried ginger rhizome (0.5–1% in the diet) for eight weeks alleviated mechanical and thermal allodynia, as well as anxiety- and depression-like behaviours and cognitive disturbances. Ginger also reduced pro-inflammatory mediators (NO, PGE_2_, TXB_2_ and IL-1β) in macrophages, and co-administration of ginger enhanced the antinociceptive efficacy of paracetamol. These findings suggest that ginger exerts anti-inflammatory and neuroprotective effects that are relevant to pain and mood symptoms associated with FM [82]. Another study using a reserpine-induced mouse model of FM found that an optimised methanolic extract of *Angelica archangelica* roots (60 °C for 36 h; containing coumarin), administered orally at 200–400 mg/kg, improved pain (higher paw withdrawal threshold), motor ability, locomotion and cognition. The extract reduced serum TNF-α and IL-1β, and attenuated brain and muscle oxidative stress (lowering TBARS and higher GSH) in a dose-dependent manner, although efficacy was limited at the lowest dose. Overall, these results indicate antinociceptive and anti-inflammatory/antioxidant properties in this preclinical FM model [83]. In a rat model of FM induced by reserpine, treatment with honokiol reduced pain, depression and anxiety, increased SOD and IL-10 levels, and decreased MDA, TNF-α and prostaglandin E_2_ (PGE_2_) levels. Honokiol also suppressed the expression of genes associated with inflammation and apoptosis. These findings imply that honokiol may have therapeutic potential in FM-like conditions [84]. In another rat model, fisetin treatment was shown to reduce oxidative stress, autophagy, apoptosis and inflammation in reserpine-induced FM. Fisetin upregulated the expression of endothelial nitric oxide synthase (eNOS) and Bcl-2, while downregulating the expression of caspase-3, microtubule-associated protein 1 light chain 3 (LC3), C/EBP homologous protein (CHOP), and TNF-α, thereby promoting structural and functional recovery in vascular and neuronal tissues [85].

From another perspective, evening primrose oil, characterised by its high γ-linolenic acid content, has been shown to exert analgesic effects in chronic pain models resembling FM, attenuating mechanical and thermal allodynia and hyperalgesia and improving anxiety- and depression-like behavioural disturbances. Furthermore, significant reductions in NO, PGE_2_, TXB2 and IL-1β levels were observed in macrophages isolated from animals fed EPO-supplemented diets. These results suggest that EPO may help to alleviate FM-like symptoms by reducing pain and inflammation, although confirmation in human FM populations is required [86].

In a two-arm randomised controlled trial including 46 women with FM, participants were allocated to either an anti-inflammatory, low-FODMAP diet (*n* = 22) or a control group that received general healthy eating recommendations (*n* = 24) for 3 months. Compared with the control group, the intervention group showed significant improvements in pain (VAS, BPI), fatigue (FSS), gastrointestinal symptoms, sleep quality (PSQI) and quality of life (FIQR, SF-36), whereas inflammatory biomarkers such as hs-CRP and ESR remained unchanged. These findings suggest that an anti-inflammatory, low-FODMAP diet may complement pharmacological treatment in alleviating FM symptoms [87]. Observational data from women with FM revealed lower serum levels of choline and leptin, and higher levels of IL-6, compared with controls. Notably, IL-6 correlated positively with pain, suggesting testable hypotheses regarding choline status and immune–neurochemical interactions in FM [88]. Additional evidence links low vitamin D status and insufficient SCFAs (particularly acetate) with higher levels of inflammatory cytokines and more severe pain. This pattern suggests that maintaining adequate vitamin D levels and consuming a diet that supports the growth of SCFA-producing beneficial bacteria may confer anti-inflammatory effects [89].

In summary, FM appears to involve an inflammatory environment characterised by elevated TNF-α, IL-6, IL-8 and IL-1β, together with altered IL-10. This inflammatory state is coupled with NLRP3–caspase-1 activation, oxidative/nitrosative stress and imbalances in lipid-derived (eicosanoids, bile acids, SCFAs) and amino acid-derived (glutamate–GABA, tryptophan–serotonin, NO) signalling pathways, all of which lower nociceptive thresholds. Table 3 summarises studies investigating the anti-inflammatory effects of phytonutrients in FM. Preclinical data suggest that several phytonutrients, such as curcumin, astaxanthin, honokiol, fisetin, *Angelica* derivatives, ginger constituents and organosulfur compounds, as well as evening primrose oil rich in γ-linolenic acid, can inhibit these pathways and improve pain- and mood-related behaviours. Although early clinical signals from anti-inflammatory/low-FODMAP dietary patterns and micronutrient/SCFA sufficiency are encouraging, they remain preliminary and support the use of such strategies as adjuncts rather than stand-alone therapies. Robust, biomarker-integrated trials using standardised, bioavailable formulations and stratifying participants by microbiome and metabolomic profiles are required to identify responders and establish clinical efficacy.

### 4.3. Microbiota Regulatory Effects

Recent studies have highlighted a significant relationship between chronic pain, particularly the pathophysiology of FM, and the gut microbiome [31,90,91]. The gut microbiome of patients with FM differs markedly from that of healthy individuals. In FM, increased relative abundances of *Clostridium*, *Bacteroides*, *Ruminococcus* and *Coprococcus* have been reported, alongside decreased levels of *Bifidobacterium*, *Lactobacillus*, *Eubacterium*, members of Lachnospiraceae family, and the phylum Firmicutes. These shifts in gut microbiota composition are accompanied by alterations in key microbial metabolites: levels of LPS, bile acids, and glutamate are increased, whereas SCFAs, tryptophan, serotonin, and gamma-aminobutyric acid (GABA) are reduced [32]. An overview of these taxa-level differences is presented in Table 4.

Patients with FM have also been shown to have higher abundances of specific bacterial species such as *Flavonifractor plautii* and *Parabacteroides merdae*, and lower abundances of other bacterial species, such as *Faecalibacterium prausnitzii*, *B. uniformis*, *Prevotella copri* and *Blautia faecis*, compared with healthy controls [33]. Several of these species, particularly *F. prausnitzii*, are known to enhance intestinal barrier function and exert antinociceptive and anti-inflammatory effects [92,93]. A recent systematic review supports these observations, reporting reduced levels of *F. prausnitzii*, Ruminococcaceae and Bifidobacteriaceae in patients with FM. This reduction may contribute to inflammation, given the central role of *F. prausnitzii* in butyrate production. Moreover, a decline in *Bifidobacterium* species has been proposed to be associated with abnormalities in central pain processing via the gut–brain axis [94].

A Mendelian randomisation study has provided evidence for a potential causal relationship between the gut microbiota and FM. Examining 119 bacterial genera, the authors reported that *Eggerthella*, *Coprococcus 2*, and *Lactobacillus* may increase the risk of FM, whereas Family XIII UCG 001 and *Olsenella* may reduce it [95]. Another study found that certain bacteria, such as *Butyricicoccus* and *Coprococcus 1*, may protect against FM, while *Eggerthella* and Ruminococcaceae *UCG-005* may increase risk. In additon, more than 80 plasma metabolites, associated with metabolic pathways, including those involving caffeine and α-linolenic acid, have been linked to FM. Specific bacterial groups were shown to influence FM risk via these metabolites. Taken together, these findings support the involvement of the gut microbiota and related metabolic pathways in FM pathophysiology and provide a rationale for exploring personalised dietary approaches, such as moderate caffeine intake, increasing omega-3 intake, adopting a low glycaemic index diet, and reducing the foods high in oxalate [40].

Differences in microbiome composition also appear to affect symptom severity. Dysbiosis has been linked in particular to patients’ pain levels, fatigue, and cognitive dysfunction [33,96]. In a study of New Zealand women, the gut microbiome of those with FM was associated with specific bacterial taxa within the Bacteroidetes phylum and several genera, including *Parabacteroides* and *Streptococcus*, which were related to pain, fatigue, cognitive impairment, migraines, poor sleep quality and gastrointestinal symptoms. Some species were also reported to affect symptoms through metabolic and neuroimmune pathways by producing secondary bile acids and neuroactive metabolites. Interestingly, an inverse association was observed between increased microbial diversity and patients’ perceptions of fatigue and energy levels, suggesting dysregulation in the gut–brain-energy axis [35]. Another study found that alterations in the gut microbiota of women with FM were associated with decreased serum α-muricholic acid levels. These changes were characterised by shifts in bile acid-metabolising species, including decreased levels of *B. uniformis*, *B. thetaiotaomicron* and *P. copri*, and increased levels of *C. scindens* and *Enterocloster bolteae*. These microbial and metabolic changes were strongly correlated with pain and fatigue severity, suggesting a role in nociceptive pain mechanisms and raising the possibility that bile acid-related markers could serve as molecular diagnostic candidates in FM [34].

**Table 4:** Summary of gut microbiota alterations in FM.

Bacterial Taxa	Change/Relationship in FM (vs. Controls)	Ref.
*Clostridium*	↑ levels in FM patients	[32]
*Bacteroides (overall)*	↑ levels in FM patients	[32]
*Ruminococcus*	↑ levels in FM patients	[32]
*Coprococcus (overall)*	↑ levels in FM patients	[32]
*Bifidobacterium*	↓ levels in FM patients	[32,94]
*Lactobacillus*	↓ levels in FM patients	[32,95]
*Eubacterium*	↓ levels in FM patients	[32]
Lachnospiraceae	↓ levels in FM patients	[32]
Firmicutes	↓ levels in FM patients	[32]
*Flavonifractor plautii*	↑ levels in FM patients	[33]
*Parabacteroides merdae*	↑ levels in FM patients	[33]
*Faecalibacterium prausnitzii*	↓ levels in FM patients; confirmed decrease in systematic review	[33,92,93,94]
*Bacteroides uniformis*	↓ levels in FM patients	[33,34]
*Prevotella copri*	↓ levels in FM patients	[33,34]
*Blautia faecis*	↓ levels in FM patients	[33]
Ruminococcaceae	↓ levels in FM patients (systematic review)	[94]
Bifidobacteriaceae	↓ levels in FM patients (systematic review)	[94]
*Eggerthella*	Higher levels may increase FM risk	[40,95]
*Coprococcus 2*	Higher levels may increase FM risk	[95]
Family XIII UCG-001	Higher levels may reduce FM risk	[95]
*Olsenella*	Higher levels may reduce FM risk	[95]
*Butyricicoccus*	Higher levels may protect against FM	[40]
*Coprococcus 1*	Higher levels may protect against FM	[40]
*Ruminococcaceae UCG-005*	Higher levels may increase FM risk	[40]
*Bacteroidetes*	Altered composition associated with FM	[35]
*Parabacteroides (overall)*	Associated with FM in New Zealand women	[35]
*Clostridium scindens*	Increased in women with FM	[34,35]
*Enterocloster bolteae*	Increased in women with FM	[34,35]
*Streptococcus* sp. *LPB022*	Associated with FM in New Zealand women	[35]
*Bacteroides thetaiotaomicron*	↓ levels in women with FM	[34]

A prospective pre–post study of 41 patients with chronic fatigue syndrome (CFS) evaluated the effects of a 10–14-month regimen of natural anti-inflammatory/antioxidative substances (NAIOSs; L-carnitine, CoQ10, taurine + lipoic acid, with or without curcumin + quercetin or N-acetyl-cysteine, plus zinc + glutamine), combined with a “leaky gut” diet (milk-free, gluten-free and low-carb diet). Initially elevated IgA/IgM responses to Gram-negative LPS (e.g., from *Pseudomonas* or *Klebsiella*) decreased significantly after treatment, and up to 24 of 41 patients showed clinical improvement or remission on the Fibromyalgia/Chronic Fatigue Scale (FF Score). Better outcomes were predicted by greater antibody attenuation, younger age, and shorter illness duration (less than 5 years). The findings support the concept of a gut-derived inflammation/LPS-translocation pathway in CFS and suggest this axis as a potential therapeutic target; however, inference is limited by the uncontrolled design [97]. Consistently, a randomised, double-blind, crossover study demonstrated that dietary substitution with Khorasan wheat beneficially modulated the gut microbiota and SCFA profile in patients with FM. The Khorasan wheat diet was associated with increased levels of beneficial bacterial groups, such as *Actinobacteria* and *Candidatus* Saccharibacteria, and higher butyric acid concentrations, alongside a decrease in the Enterococcaceae family. These microbiota changes were positively correlated with improvements in FM symptoms, including fatigue, widespread pain, and sleep quality [98]. A study evaluating the efficacy of probiotic and prebiotic supplements in women with FM found that providing a total of 4 × 10^10^ CFU/day of four strains (*L. acidophilus* L1 (2.9 × 10^9^), *L. rhamnosus* liobif (2.9 × 10^9^), *B. longum* (2.9 × 10^9^) and *Saccharomyces boulardii* (1.3 × 10^9^)) significantly improved pain, sleep quality, depression and anxiety over eight weeks. In contrast, prebiotic supplementation (10 g/day inulin) only improved pain and sleep quality. These findings suggest that probiotics may be beneficial in reducing FM symptoms [99].

A pilot randomised controlled trial evaluated the effect of an eight-week multi-probiotic supplement containing *L. rhamnosus GG*, *L. paracasei*, *L. acidophilus* and *B. bifidum* on cognitive function in patients with FM. Probiotic supplementation improved attention performance but did not significantly affect memory [100]. Another randomised, double-blind, placebo-controlled study assessed the efficacy and tolerability of the multi-strain probiotic VSL#3^®^ (450 billion CFU/sachet) for gastrointestinal symptoms associated with FM. VSL#3^®^ contains the following strains: *Streptococcus thermophilus* BT01; *B. breve* BB02; *B. animalis* subsp. *lactis* BL03; *B. animalis* subsp. *lactis* BI04; *L. acidophilus* BA05; *L. plantarum* BP06; *L. paracasei* BP07; and *L. helveticus* BD08. After 12 weeks of probiotic or placebo administration, no significant differences were observed between groups in abdominal pain, bloating, or flatulence. However, among participants who responded to the probiotic, symptom improvement appeared to be sustained for a longer period [101]. Conversely, an animal study investigated a potential mechanism relevant to conditions characterised by elevated blood glutamate levels, such as FM and chronic pain syndromes. The *B. adolescentis* IPLA60004 strain, which can convert glutamate to GABA, was administered to healthy mice for 14 days. *B. adolescentis* IPLA60004 significantly reduced serum glutamate levels, although changes in GABA levels were sex-dependent. While no FM model was employed, these results imply that probiotics that GABA-producing probiotics might have therapeutic potential in chronic pain conditions in which excess glutamate contributes to symptom generation [102].

In one noteworthy study, faecal microbiota transplantation (FMT) from individuals with FM resulted in a persistent increase in pain sensitivity in germ-free mice [90]. A clinical trial evaluating FMT in patients with FM reported significant improvements in pain, anxiety, depression and sleep disturbance scores from the second month onwards. By six month, serotonin and GABA levels had increased, whereas glutamate levels had decreased [103]. Taken together, these data suggest that FMT may represent a promising strategy for alleviating FM symptoms and modulating neurotransmitter balance, although confirmatory trials with larger samples and rigorous controls are still needed

Although alterations in the microbiota of individuals with FM and their associations with symptom severity have been reported, the current evidence is limited by small sample sizes, heterogeneous methodologies, and the absence of robust causal inference at the clinical level. Microbiota-targeted strategies such as probiotics, prebiotics, personalised diets, and FMT have been proposed as potential approaches to alleviate FM symptoms. However, due to methodological limitations and the preliminary nature of the existing data, these strategies cannot yet be recommended as standard treatment. Nevertheless, personalised nutritional counselling and microbiota-informed approaches may be considered as adjuncts to conventional management, given their generally low risk when appropriately supervised. A summary of studies evaluating gut microbiota alterations and microbiota-targeted interventions in FM is presented in Table 5.

### 4.4. Effects on SIRT1

Mechanistically, SIRT1 acts as a nodal regulator that links redox balance, metabolic status and inflammatory signalling to nociplastic pain. Experimental and clinical studies indicate that lower SIRT1 activity is associated with heightened pain sensitivity. In contrast, increased SIRT1 signalling has been shown to have antinociceptive, anti-inflammatory and antioxidant effects in chronic pain models, including those related to fibromyalgia [7,9,10,11,104,105]. SIRT1 enhances mitochondrial and antioxidant defences by deacetylating key transcription factors and signalling molecules (e.g., PGC-1α, FOXO and MnSOD) [106,107,108,109,110,111,112,113], while also dampening the expression of pro-inflammatory cytokines such as TNF-α, IL-1β and IL-6 driven by NF-κB [41,42,114,115,116]. These actions are highly relevant to FM, a condition characterised by the coexistence of oxidative stress, low-grade inflammation and central sensitisation.

Polyphenols such as resveratrol, fisetin, quercetin and curcumin have been shown to modulate SIRT1 signalling in cells, animals and a limited number of humans, resulting in improvements to markers of oxidative stress, metabolism and neuroprotection [117,118,119,120]. However, these data largely come from non-fibromyalgia contexts, so SIRT1-mediated effects in FM remain mechanistically plausible but not yet demonstrated in patients. In one rat study using a FM model, the polyphenol compound miristic acid activated SIRT1, enhanced antioxidant defences (increased Nrf2 and HO-1), suppressed inflammatory signalling (decreased NLRP3, IL-1β and NF-κB) and modulated apoptosis-related proteins (BAX/Bcl-2 ratio) [7]. Another recently published in vivo study demonstrated that the bergamot polyphenolic fraction (BPF) reduces pain and oxidative stress by regulating SIRT1 activity in rats exhibiting inflammation and hyperalgesia. BPF intervention preserved SIRT1 activity and reduced the levels of the oxidative stress markers MDA and 8-hydroxy-2′-deoxyguanosine (8-OHdG). It also decreased nitration and other post-translational modifications in proteins. BPF intervention also alleviated hyperalgesia and allodynia. These research findings suggest that polyphenols may be useful in managing inflammation- and oxidative stress-induced pain by activating SIRT1 [104]. Similarly, a mitochondria-targeted antioxidant molecule reduced pain and improved locomotor and depressive-like behaviours in animals by enhancing SIRT1-mediated antioxidant enzyme activities (SOD and CAT), suppressing proinflammatory markers (TNF-α and NF-κB) and modulating apoptotic pathways (decreased BAX, increased Bcl-2) [105].

In summary, SIRT1 emerges as a plausible integrative node linking oxidative stress, mitochondrial dysfunction, metabolism and inflammation to nociceptive sensitisation in FM. From a mechanistic perspective, SIRT1 deacetylates NF-κB, FOXO and p53, thereby reducing the transcription of pro-inflammatory cytokines (e.g., IL-1, TNF-α, IL-6 and IL-8) and increasing the expression of antioxidant defences (SOD2/MnSOD and CAT) and mitochondrial biogenesis programmes (PPARs/NRF and TFAM). Table 6 summarises studies investigating the modulation of the SIRT1 pathway by phytonutrients in FM and pain models, and the associated therapeutic outcomes. Preclinical studies further suggest that polyphenols (e.g., resveratrol, quercetin, curcumin and fisetin) and related interventions can activate SIRT1, reduce oxidative and nitrosative stress, and alleviate NLRP3-associated neuroinflammation. These interventions can also improve behaviours related to pain and mood. However, there is still limited and heterogeneous human evidence. Taken together, these in vivo findings support a biologically plausible role for SIRT1 activation by polyphenols in pain modulation, but their translational relevance to FM remains uncertain and requires confirmation in human trials incorporating SIRT1-related biomarkers.

## 5. Diet Models High in Phytonutrient Content and FM

Recent scientific publications emphasise that nutrition is a fundamental factor in the treatment of FM, which is a condition that causes widespread muscle pain and fatigue [121,122]. Observational studies report that adults with FM consume higher quantities of ultra-processed foods (UPFs) and have higher dietary inflammatory index (DII) scores than healthy individuals. Furthermore, this group was found to have a higher body mass index (BMI) and fat mass, as well as lower muscle mass, and experience more severe pain and fatigue. Low intakes of magnesium, vitamin C and polyphenols, and high intakes of saturated fat and refined carbohydrates were also observed [123]. Cross-sectional data further suggest that diets with higher antioxidant potential may be associated with lower oxidative stress in FM [73,124]. A narrative ‘neuro-nutritional’ review has indicated that nutritional interventions such as vitamin D, magnesium, vitamin B12, coenzyme Q10, probiotics, omega-3 fatty acids, melatonin, S-adenosylmethionine, acetyl-L-carnitine, and curcumin may improve FM symptoms [125]. In a rat model of cobra venom-induced trigeminal neuralgia, curcumin (45 mg/kg twice daily for 28 days) alleviated pain behaviours and improved spatial memory by restoring neuronal integrity in the hippocampus. While these findings originate from a neuropathic pain model, they suggest that curcumin’s neuroprotective and antinociceptive actions may also be applicable to fibromyalgia-related pain and cognitive dysfunction [126].

A narrative review of nutritional interventions in FM reported that consuming unprocessed grains, olive oil, low-energy foods, vegetarian foods, low-FODMAP foods, gluten-free foods, foods free of monosodium glutamate and aspartame, and foods based on the Mediterranean diet can significantly improve chronic pain, anxiety, depression, cognitive function, sleep patterns and gastrointestinal symptoms [127]. A randomised, controlled clinical trial involving 31 women with FM evaluated the effectiveness of a two-month intervention comprising an olive-tree-based supplement and a gluten-free, low-FODMAP, low-histamine diet (IGUBAC-Diet^®^) versus usual diet. Participants were allocated to the intervention or control condition and assessed at baseline and after 8 weeks. Compared with baseline and with the control group, the IGUBAC-Diet^®^ arm showed reductions in Chronic Pain Grade and pain catastrophising, an improvement in Fatigue Severity Scale scores, but no change in the Fatigue Impact Scale; overall symptom frequency also decreased [77,128]. However, these findings should be interpreted with caution, given the small sample size, brief follow-up period, and limited blinding and control procedures. An eight-week intervention study showed that a personalised Mediterranean diet improved clinical parameters such as fatigue, anxiety and functional limitations in patients with FM. Unlike the traditional Mediterranean diet, this one excluded dairy products and eggs. These results suggest that poor dietary habits may worsen the progression of the disease, and that following an anti-inflammatory dietary plan can enhance quality of life [129]. Consistently, a recently published systematic review evaluated the potential beneficial effects of the Mediterranean diet’s antioxidant, anti-inflammatory and low-antigen properties on FM symptoms. The review demonstrated that the diet may alleviate chronic widespread pain, autonomic dysfunction, persistent fatigue, sleep disorders and cognitive impairments, such as fibro fog [130].

Taken together, these observational and interventional data suggest, but do not conclusively prove, that dietary patterns rich in plant-based foods, antioxidants and fibre may support FM management. Weight loss and improvements in body composition have been associated with reduced inflammation and better quality of life, raising the possibility that changes in adiposity and psychosocial factors partly mediate dietary effects [127]. A recent systematic review of 12 trials involving 546 participants found that nine dietary interventions, including all three plant-based diets, were associated with statistically significant improvements in pain outcomes. However, due to the small sample sizes and short durations of the trials, the authors concluded that the evidence was insufficient to recommend any specific diet for FM and that further high-quality trials were needed [122]. Moreover, it is emphasised that pro-inflammatory dietary profiles may worsen FM symptoms and that anti-inflammatory nutrition strategies may offer potential benefits in multidisciplinary FM management [123].

## 6. Conclusions

Fibromyalgia is a biologically complex syndrome involving oxidative stress, low-grade systemic inflammation, alterations in the gut microbiota and dysregulation of the SIRT1-mediated signalling axis. This review compiles evidence that phytonutrients such as polyphenols (e.g., resveratrol, quercetin, fisetin and anthocyanins), carotenoids, glucosinolates and organo-sulphur compounds exert antioxidant, anti-inflammatory and neuromodulatory effects. They may also target core pathophysiological mechanisms of FM by supporting SIRT1 activation, thereby influencing oxidative stress, inflammation, neurotransmitter imbalance and mitochondrial dysfunction. Given the link between reduced microbiota-derived metabolites (particularly SCFAs) and heightened pain sensitivity and neuroimmune imbalance, phytonutrient-rich, plant-based dietary patterns have significant potential to address both symptom clusters and underlying pain biology. However, the heterogeneous designs, small sample sizes and short follow-up periods of supplement-focused studies limit the generalisability of the current evidence. Accordingly, phytonutrient interventions should be regarded as a complementary component of multidisciplinary management, rather than replacements for guideline-based pharmacotherapy.

In clinical practice, priority should be given to food-based strategies that permanently improve the overall diet quality. Adopting a Mediterranean-style, plant-based diet comprising a variety of vegetables and fruits from different colour groups, whole grains, legumes, olive oil, and regularly consuming oily seeds can increase phytochemical intake and has been associated with reduced chronic disease risk and improved cardiometabolic and cognitive outcomes [38,39,43,44,45,46,51,54,127,130]. Reducing ultra-processed foods, refined carbohydrates, and saturated fats can also help to reduce oxidative stress and inflammatory load [123]. In this context, regular consumption of anthocyanin-rich red–purple fruits, cruciferous vegetables containing glucosinolates, carotenoid-rich orange–yellow vegetables and allium vegetables containing organo-sulphur compounds represents a pragmatic way to increase dietary phytonutrient density [37,38,42,43,44,45,50]. Increasing fibre intake and judiciously using fermented foods can also support microbiota diversity and SCFA production, with potential benefits along the gut–brain axis.

A cautious and individualised approach to supplements is recommended. Options such as curcumin, selected polyphenols, coenzyme Q10, L-carnitine or multi-strain probiotics may be considered as an adjunct to a food-based intervention in selected patients, based on time-dependent response monitoring and careful consideration of accompanying symptom clusters (e.g., pain, fatigue, sleep and gastrointestinal complaints) and potential drug interactions. In line with “food-first” principles, high-dose or multi-supplement regimens should be avoided without specialist supervision, and supplements should not be positioned as substitutes for dietary modification or standard FM care. In clinical practice, systematically monitoring pain (VAS/BPI), fatigue (FSS), sleep (PSQI), quality of life (FIQR/SF-36) and gastrointestinal symptom scores at baseline and after 8–12 weeks, identifying responders and dynamically adapting the nutrition plan will increase real-world effectiveness. Given the multidimensional nature of FM, the integration of dietary interventions with exercise prescriptions, psychosocial approaches, and pharmacotherapy when necessary is critically important.

## 7. Limitations

The findings of this review should be interpreted in light of several important limitations. First, human studies are heterogeneous, with small sample sizes and short follow-up periods. Outcome measures (e.g., pain, FIQR, PSQI, and gastrointestinal symptom scores) and biomarker panels (e.g., MDA/4-HNE, SOD/CAT/GPx, PON-1/NO, OSI, and cytokines) are not standardised. Second, although evidence from animal studies is mechanistically consistent, its clinical translatability is limited. Variability in dose and formulation (e.g., curcumin bioavailability forms, probiotic strains, CFU, and duration), as well as treatment adherence, makes it difficult to determine effect sizes. Third, relationships in the microbiome field are largely observational, and broader, well-controlled data are required to establish causality and to ensure the safety of interventions such as Mendelian randomisation studies and FMT. Fourth, publication bias, language and time restrictions, and inadequate control of confounders such as diet, physical activity, BMI, comorbidities, and medications in most studies reduce the certainty of inferences. Finally, human data on the SIRT1 axis in FM are limited, with most inferences based on indirect biomarkers and animal models. Overall, the available evidence for symptom improvement with dietary patterns (e.g., Mediterranean-type diets, increased fibre, fermented foods, and reduced processed food) comes from a small number of heterogeneous trials and should be considered preliminary. Findings for single supplements (e.g., curcumin, other polyphenols, CoQ10 and multi-strain probiotics) are mixed and inconsistent, and existing RCTs of ALA have not demonstrated clinical benefit.

## 8. Research Gaps and Future Perspectives

The research agenda should shift towards longer-term, well-phenotyped randomised controlled trials that prioritise the food matrix and integrate mechanistic endpoints. These should jointly assess SIRT1 activity, NRF2-related redox pathways, oxidative stress and cytokine panels, and SCFAs and other microbiota-derived metabolites alongside clinical outcomes, ideally within multi-omic frameworks of the diet–microbiota–metabolome axis. Systematic testing is required to investigate gender-specific differences, pain-GI-sleep dominant sub-phenotypes, dose–response relationships, and the effects of preparation and cooking techniques on bioavailability. Finally, public health policies that aim to reduce UPF consumption and focus on feasibility dimensions such as compliance, accessibility, cost and cultural appropriateness will support the sustainability of clinical gains at the population level. Within this holistic framework, a phytonutrient-rich, microbiota-friendly dietary approach emerges as a biologically robust and clinically feasible option for complementing FM management.

## Figures and Tables

**Table 1:** Summary of database search parameters, eligibility criteria and exemplar keywords.

Component	Description
Databases	PubMed, Web of Science, Scopus, ScienceDirect
Time period	1 January 2005–16 October 2025
Languages	Full text in English or Turkish
Study types included	Human studies and animal models
Study types excluded	In vitro studies, editorials, reviews without primary data, grey literature, preprints
Population/condition	Fibromyalgia or fibromyalgia-related pain models
Intervention/exposure	Phytonutrient-rich diets or isolated phytonutrients (polyphenols, carotenoids, etc.)
Mechanisms of interest	Oxidative stress, inflammation, gut microbiota, SIRT1 signalling
Example keywords/strings	“fibromyalgia AND polyphenols”, “fibromyalgia AND Mediterranean diet”, “SIRT1 AND pain”, etc.

**Table 2:** Summary of Studies Investigating the Effects of Phytonutrients on Oxidative Stress Mechanisms in FM.

Study Focus	Population/Model	Study Design	Main Findings	Reference
Fasting plasma vitamins (A, E, C, β-carotene) & oxidative markers (LP, NO)	FM patients vs. healthy controls	Case–control	FM vs. age-matched controls: ↓plasma vitamins A & E, ↑lipid peroxidation (LP); ↔ vitamin C, β-carotene, NO— consistent with altered fat-soluble antioxidant status and oxidative damage; case–control, single time point, small n → limited causality	[59]
Vitamin C + E (VCE) ± Exercise (EX)	FM patients	Controlled clinical pilot study	Lipid peroxidation ↓, vitamins A/E ↑, GSH ↑, erythrocyte GPx ↑, β-carotene ↔; no improvement in FM symptoms	[65]
Anti-IO&NS nutraceuticals (NAIOS)	ME/CFS patients	Prospective care-as-usual, pre–post observational	Marked reductions in IgM autoimmunity to OSEs and NO-adducts; greater falls in OSE-IgM correlated with lower FM and Fatigue scores	[64]
Resveratrol and rice oil supplementation	Reserpine-induced mice model of FM	Animal study	Reduced pain behaviors, lowered CSF reactive species, antidepressant-like effects, efficacy comparable to pregabalin	[67]
Oxidant–antioxidant balance (TAC, SOD, CAT, GPx)	FM patients vs. healthy controls	Case–control	TAC, SOD, CAT, GPx levels reduced in FM; increased oxidative stress inversely correlated with FIQR	[30,57]
Lutein and β-carotene nanodispersions	Reserpine-induced rat model of FM	Animal study	↓ MDA, H_2_O_2_, NO; ↑ GSH → restored oxidative balance	[70]
Alpha-lipoic acid (ALA) supplementation	FM patients	Randomised, placebo-controlled, crossover trial	No significant effect on pain, FIQ, sleep, or mood; subgroup benefit in males only	[66]
Oxidative stress and muscle performance	FM patients	Observational	High TBARS and low SOD levels were associated with increased muscle pain, reduced lean mass, and poorer quality of life	[25]
Micronutrient and plant-based supplementation	FM patients	Systematic review	C vitamin, CoQ10, ferric carboxymaltose, *Nigella sativa*, L-carnitine, Sun Chlorella™ → associated with pain reduction	[73]
PON-1 activity and MDA levels	FM patients vs. healthy controls (Turkish)	Case–control	Reduced PON-1 activity and elevated MDA correlated with higher pain, FIQ, and depression scores	[62]
Gallic acid administration	CCI + CUMS pain–depression model (rats)	Animal study	Regulated P2X7–ROS signaling; reduced iron accumulation and mitochondrial damage; inhibited microglial ferroptosis	[71]
Serum MDA, CRP, and micronutrient levels	FM patients vs. healthy controls (Bangladesh)	Case–control	Higher MDA and CRP; lower vitamin C, calcium, magnesium, zinc, and copper in FM → associated with oxidative stress and micronutrient imbalance	[60]
Oxidative stress markers (TAC, TOC, MDA, OSI)	FM patients vs. healthy controls (Turkish)	Case–control	Elevated oxidative stress parameters (TAC, TOC, MDA, and OSI levels); no significant correlation with symptom severity or psychological scores	[61]
NRF2 and NFκB modulation in fibromyalgia	Intermittent cold stress (ICS)-induced FM mice model	Animal study	4-APSB (1 mg/kg) reduced nociceptive and depressive-like behaviors; normalized TBARS; downregulated Nrf2 and NFκB expression; restored Na^+^/K^+^-ATPase activity and redox balance in central nervous system, indicating antioxidant and antinociceptive potential	[58]
Isorhamnetin administration	Reserpine-induced rat model of FM	Animal study	↑ GSH, ↓ TBARS, ↓ TNF-α & IL-1β; ↑ serotonin, ↑ norepinephrine ↓ glutamate → reduced pain and depression-like behaviors, improved cognition	[68]
Anthocyanin supplementation (200 mg/kg)	Reserpine-induced rat model of FM	Animal study	↑ Serotonin, ↑ miR-145-5p, ↑ miR-451a ↓ H_2_O_2_, ↓ TNF-α & caspase-3 → improvement in pain, depression, and cognitive impairment	[69]

**Table 3:** Summary of Studies Investigating the Anti-Inflammatory Effects of Phytonutrients in Fibromyalgia.

Study Focus	Sample/Model	Study Design	Key Findings	Reference
Curcumin intervention	Reserpine-induced rat model of pain–depression dyad	Animal study	At 100–300 mg/kg (i.p.), dose-dependently improved nociceptive threshold and reduced depressive behavior; restored dopamine, norepinephrine, and serotonin levels; decreased substance P, nitrodative stress, inflammatory cytokines (TNF-α, IL-1β), NF-κB, and caspase-3 in cortex and hippocampus	[79]
The effects of dietary evening primrose oil (EPO) on chronic pain and inflammatory status	Mice exposed to intermittent cold stress (ICS)	Animal study	EPO supplementation reduced mechanical and thermal allodynia and hyperalgesia, improved anxiety- and depression-like behaviors, and decreased NO, PGE_2_, TXB_2_, and IL-1β levels in macrophages	[86]
Dried ginger, and/or paracetamol intervention	ICS-induced mouse model of fibromyalgia	Animal study	↓mechanical/thermal allodynia; ↓mechanical hyperalgesia; ↑performance in anxiety/depression-like assays; macrophages: ↓NO, ↓PGE_2_, ↓TXB_2_, ↓IL-1β; ginger + paracetamol > paracetamol alone—consistent with an antinociceptive/anti-inflammatory axis	[82]
*Angelica archangelica* root extract (optimized methanolic) in FM	Reserpine-induced mice model of FM	Animal study	At 200–400 mg/kg, improved pain (higher paw-withdrawal threshold), motor ability, locomotion, and cognition; reduced serum TNF-α and IL-1β; attenuated oxidative stress in brain/muscle (lower TBARS, higher GSH); dose-dependent effects	[83]
Cytokine profiles in FM (TNF-α, IL-6, IL-8, IL-10)	FM patients	Systematic review	FM associated with elevated pro-inflammatory cytokines (TNF-α, IL-6, IL-8) and altered anti-inflammatory IL-10; supporting role of chronic low-grade inflammation in symptom expression	[27]
Fisetin intervention	Reserpine-induced rat model of FM	Animal study	FM associated with excessive autophagy, endothelial/vascular injury, and apoptosis; gene pattern: ↓eNOS/Bcl-2, ↑caspase-3/LC3/BECN1/CHOP/TNF-α; fisetin partly reverses these—supporting an oxidative-stress–autophagy–apoptosis axis	[85]
Anti-inflammatory and low-FODMAP diet	FM patients (RCT)	Randomised controlled trial	Significant improvement in pain (VAS, BPI), fatigue (FSS), GI symptoms, sleep (PSQI), and quality of life (FIQR, SF-36); no significant changes in hs-CRP or ESR	[87]
NLRP3 inflammasome inhibition by astaxanthin	Reserpine-induced mice model of FM	Animal study	Astaxanthin downregulated NLRP3 expression; suppressed pro-inflammatory cytokines (TNF-α, IL-6, IL-1β), improved the level of IL-10, mechanical and thermal hyperalgesia, sleep disturbances, and depressive-like symptoms	[78]
Honokiol treatment	Reserpine-induced rat model of FM	Animal study	↓ TNF-α, ↓ PGE_2_, ↓ MDA, ↑ IL-10, ↑ SOD; suppressed inflammation- and apoptosis-related gene expression; reduced pain, depression, and anxiety	[84]
Choline intake and IL-6 levels	FM women vs. healthy controls	Case–control	Lower serum choline and leptin; higher IL-6; positive correlation between IL-6 and pain scores → suggesting role of dietary choline deficiency in inflammation	[88]
Vitamin D and SCFA levels in inflammation	FM women	Observational	Low vitamin D and SCFA (especially acetate) associated with elevated pro-inflammatory cytokines and more severe pain → vitamin D and SCFA support may confer anti-inflammatory benefits	[89]

**Table 5:** Summary of Studies Investigating the Gut Microbiota Alterations and Microbiota-Targeted Interventions in Fibromyalgia.

Study Focus	Sample/Model	Study Design	Key Findings	Reference
NAIOS + “leaky-gut” diet targeting LPS-related immune activation	CFS patients	Prospective pre–post (uncontrolled)	Significant declines in IgM to LPS from *Hafnia alvei*, *Pseudomonas aeruginosa*, *Morgenella morganii*, *Pseudomonas putida*, *Citrobacter koseri*, *Klebsiella pneumoniae*; peak IgM, peak IgA, and combined peaks fell. Up to 24/41 showed clinical improvement/remission; greater antibody reductions predicted lower FF scores	[97]
Specific taxa differences	FM patients vs. controls	Observational	↑ *Flavonifractor plautii*, *Parabacteroides merdae*; ↓ *Faecalibacterium prausnitzii*, *Bacteroides uniformis*, *Prevotella copri*, *Blautia faecis*; ↓ *F. prausnitzii* linked to impaired barrier and inflammation	[33]
Multi-strain probiotic (4 strains)	FM patients	Pilot Randomised controlled trial	Improved attention performance; no significant change in memory	[100]
VSL#3^®^ probiotic (8 strains)	FM with GI symptoms	Randomised controlled trial	No significant difference vs. placebo in abdominal pain/bloating; responders maintained improvement longer	[101]
Kamut^®^ (Khorasan wheat) intervention	FM patients	Randomised, double-blind, crossover	↑ *Actinobacteria*, *Candidatus* Saccharibacteria, ↑ butyrate; ↓ Enterococcaceae; improved fatigue, pain, and sleep quality	[98]
Mendelian randomization	Genetic datasets (n = 119 genera)	MR analysis	*Eggerthella*, *Coprococcus2*, *Lactobacillus* → ↑ FM risk; FamilyXIIIUCG001, *Olsenella* → ↓ risk	[95]
Bile acid–related microbiota changes	FM women	Observational	↓ *B. uniformis*, *B. thetaiotaomicron*, *P. copri*; ↑ *C. scindens*, *E. bolteae*; ↓ serum α-muricholic acid correlated with pain and fatigue severity	[34]
Microbiota composition in FM	FM patients vs. healthy controls	Observational	↑ *Clostridium*, *Bacteroides*, *Ruminococcus*, *Coprococcus*; ↓ *Bifidobacterium*, *Lactobacillus*, *Eubacterium*, Lachnospiraceae, Firmicutes; associated with altered metabolites (↑ LPS, bile acids, glutamate; ↓ SCFA, serotonin, tryptophan, GABA)	[32]
Probiotic vs. prebiotic supplementation	FM women	Randomised controlled trial	Probiotic (*L. acidophilus*, *L. rhamnosus*, *B. longum*, *S. boulardii*): improved pain, sleep, depression, anxiety; Prebiotic (inulin): improved pain, sleep only	[99]
FMT in FM patients	FM patients	Clinical study	After 2 months: ↓ pain, anxiety, depression, sleep disturbance; after 6 months: ↑ serotonin, GABA; ↓ glutamate; suggesting neuromodulatory benefits	[103]
Systematic review of gut taxa	FM patients	Systematic review	Decreased *F. prausnitzii*, Ruminococcaceae, Bifidobacteriaceae; low butyrate production may contribute to inflammation and central sensitization	[94]
Metabolomic–microbiota associations	FM patients	Observational	>80 plasma metabolites linked to FM (e.g., caffeine, α-linolenic acid metabolism); diet–microbiota–metabolite interactions suggest benefit of tailored diets (↑ omega-3, ↓ caffeine, ↓ oxalate)	[40]
Microbiota diversity and symptoms	FM women (New Zealand)	Observational	Specific taxa (*Bacteroidetes*, *Parabacteroides*, *Clostridium scindens*, *Enterocloster bolteae*, *Streptococcus sp.*) associated with pain, fatigue, cognition, sleep, GI symptoms; higher diversity paradoxically linked to more fatigue	[35]
Fecal microbiota transplantation (FMT)	Germ-free mice transplanted with FM microbiota	Animal model	FM-derived microbiota induced persistent pain hypersensitivity	[90]

**Table 6:** Summary of Studies Investigating SIRT1 Pathway Modulation by Phytonutrients and Related Therapeutic Implications in FM and Pain Models.

Study Focus	Sample/Model	Study Design	Key Findings	Reference
Myrisitrin (polyphenol)	Reserpine-induced rat model of FM	Experimental intervention	↑ SIRT1, ↑ Nrf2, ↑ HO-1; ↓ NLRP3, ↓ IL-1β, ↓ NF-κB; balanced BAX/Bcl-2 ratio → antioxidant, anti-inflammatory, anti-apoptotic effects	[7]
Bergamot Polyphenolic Fraction (BPF)	Hyperalgesia and inflammation rat model	Nutraceutical; polyphenol mix	Preserved SIRT1 activity; ↓ MDA, ↓ 8-OHdG, ↓ nitrosative stress; ↓ allodynia and hyperalgesia	[104]
Mitochondria-targeted antioxidant molecule	Reserpine-induced rat model of FM	Pharmacological compound	↑ SIRT1-mediated SOD and CAT activity; ↓ TNF-α, ↓ NF-κB, ↓ apoptosis (↓ BAX, ↑ Bcl-2); improved motor and mood function	[105]

## Supplementary Materials

The following supporting information can be downloaded at: https://www.mdpi.com/article/10.3390/medicina61122211/s1, Table S1: Full database search strings, Table S1B: Data-charting form, Section S1C: Deduplication rules, Section S1D: Controlled exclusion reasons.
