# Supplementary material for: Dietary Phytonutrients in Fibromyalgia: Integrating Mechanisms, Biomarkers, and Clinical Evidence—A Narrative Review
Source: Medicina (Kaunas). 2025 Dec 15;61(12):2211. doi: 10.3390/medicina61122211 (PMC12734507; doi:10.3390/medicina61122211)
Supplement: Supplementary file 1 [file medicina-61-02211-s001.zip › medicina-3990074-supplementary.pdf]

Supplementary File

Search strategy, strings, and screening fields

Coverage: database inception to 16 October 2025; only English studies were considered. Google Scholar was used only for forward citation tracking (first ~200 relevant results). Grey literature and preprints were not included. Records were deduplicated in Mendeley.

Table S1. Full database search strings

**PubMed (Title/Abstract)**		
**Database**	Fields / Mode	Full Boolean search string
**PubMed**	Title/Abstract	("fibromyalgia"[Title/Abstract] OR fibromyalg*[Title/Abstract]) AND (phytonutrient*[Title/Abstract] OR phytochemical*[Title/Abstract] OR polyphenol*[Title/Abstract] OR flavonoid*[Title/Abstract] OR carotenoid*[Title/Abstract] OR glucosinolate*[Title/Abstract] OR organosulfur[Title/Abstract] OR organosulphur[Title/Abstract] OR resveratrol[Title/Abstract] OR quercetin[Title/Abstract] OR curcumin[Title/Abstract] OR fisetin[Title/Abstract] OR anthocyanin*[Title/Abstract] OR sulforaphane[Title/Abstract] OR garlic[Title/Abstract]) AND ("oxidative stress"[Title/Abstract] OR antioxidant*[Title/Abstract] OR inflammation[Title/Abstract] OR cytokine*[Title/Abstract] OR "NF-κB"[Title/Abstract] OR NF-kB[Title/Abstract] OR NLRP3[Title/Abstract] OR SIRT1[Title/Abstract] OR sirtuin*[Title/Abstract] OR microbiota[Title/Abstract] OR dysbiosis[Title/Abstract] OR "short-chain fatty acid*"[Title/Abstract] OR SCFA[Title/Abstract] OR butyrate[Title/Abstract] OR "bile acid*"[Title/Abstract] OR glutamate[Title/Abstract] OR GABA[Title/Abstract])
**PubMed (MeSH + Title/Abstract)**		
**Database**	Fields / Mode	Full Boolean search string
**PubMed**	MeSH + Title/Abstract	("Fibromyalgia"[MeSH] OR fibromyalg*[Title/Abstract]) AND ("Phytochemicals"[MeSH] OR "Plant Preparations"[MeSH] OR polyphenols[MeSH] OR carotenoids[MeSH] OR glucosinolates[MeSH] OR "Organosulfur Compounds"[MeSH] OR resveratrol[MeSH] OR curcumin[MeSH] OR (quercetin[Title/Abstract] OR fisetin[Title/Abstract] OR anthocyanin*[Title/Abstract] OR sulforaphane[Title/Abstract] OR garlic[Title/Abstract])) AND ("Oxidative Stress"[MeSH] OR oxidative stress[Title/Abstract] OR antioxidants[MeSH] OR "Inflammasomes"[MeSH] OR NLRP3[Title/Abstract OR "NF-kappa B"[MeSH] OR NF-kB[Title/Abstract] OR "Sirtuin 1"[MeSH] OR SIRT1[Title/Abstract] OR "Gastrointestinal Microbiome"[MeSH] OR microbiota[Title/Abstract] OR dysbiosis[Title/Abstract] OR "Short-Chain Fatty Acids"[MeSH] OR butyrate[Title/Abstract] OR "Bile Acids and Salts"[MeSH] OR "Gamma-Aminobutyric Acid"[MeSH] OR GABA[Title/Abstract] OR glutamate[Title/Abstract])
**PubMed (MeSH + Designation/Abstract)**		
**Database**	Fields / Mode	Full Boolean search string
**PubMed**	MeSH + Title/Abstract	("Fibromyalgia"[MeSH] OR fibromyalg*[Title/Abstract]) AND ("Phytochemicals"[MeSH] OR "Plant Preparations"[MeSH] OR polyphenols[MeSH] OR carotenoids[MeSH] OR glucosinolates[MeSH] OR "Organosulfur Compounds"[MeSH] OR resveratrol[MeSH] OR curcumin[MeSH] OR (quercetin[Title/Abstract] OR fisetin[Title/Abstract] OR anthocyanin*[Title/Abstract] OR sulforaphane[Title/Abstract] OR garlic[Title/Abstract])) AND ("Oxidative Stress"[MeSH] OR oxidative stress[Title/Abstract] OR antioxidants[MeSH] OR "Inflammasomes"[MeSH] OR NLRP3[Title/Abstract OR "NF-kappa B"[MeSH] OR NF-kB[Title/Abstract] OR "Sirtuin 1"[MeSH] OR SIRT1[Title/Abstract] OR "Gastrointestinal Microbiome"[MeSH] OR microbiota[Title/Abstract] OR dysbiosis[Title/Abstract] OR "Short-Chain Fatty Acids"[MeSH] OR butyrate[Title/Abstract] OR "Bile Acids and Salts"[MeSH] OR "Gamma-Aminobutyric Acid"[MeSH] OR GABA[Title/Abstract] OR glutamate[Title/Abstract])

Table S1. *Cont.*

**Web of Science Core Collection**		
**Database**	Fields / Mode	Full Boolean search string
**Web of Science**	TS (Topic)	TS=(fibromyalgia OR fibromyalg*) AND TS=(phytonutrient* OR phytochemical* OR polyphenol* OR flavonoid*OR carotenoid* OR glucosinolate* OR organosulfur OR organosulphur OR resveratrol OR quercetin OR curcumin OR fisetin OR anthocyanin* OR sulforaphane OR garlic) AND TS=("oxidative stress" OR antioxidant* OR inflammation OR cytokine* OR "NF-κB" OR "NF-kB" OR NLRP3 OR SIRT1 OR sirtuin* OR microbiota OR dysbiosis OR "short-chain fatty acid*" OR SCFA OR butyrate OR "bile acid*" OR glutamate OR GABA)<br>Indexes: SCI-EXPANDED, SSCI, ESCI
**Scopus**		
**Database**	Fields / Mode	Full Boolean search string
**Scopus**	TITLE-ABS-KEY	(TITLE-ABS-KEY(fibromyalgia OR fibromyalg*)) AND (TITLE-ABS-KEY(phytonutrient* OR phytochemical* OR polyphenol* OR flavonoid* OR carotenoid* OR glucosinolate* OR organosulfur OR organosulphur OR resveratrol OR quercetin OR curcumin OR fisetin OR anthocyanin* OR sulforaphane OR garlic)) AND (TITLE-ABS-KEY("oxidative stress" OR antioxidant* OR inflammation OR cytokine* OR "NF-κB" OR "NF-kB" OR NLRP3 OR SIRT1 OR sirtuin* OR microbiota OR dysbiosis OR "short-chain fatty acid*" OR SCFA OR butyrate OR "bile acid*" OR glutamate OR GABA))
**ScienceDirect**		
**Database**	Fields / Mode	Full Boolean search string
**ScienceDirect**	Title/Abstract/Keywords	("fibromyalgia" OR fibromyalg*) AND (phytonutrient* OR phytochemical* OR polyphenol* OR flavonoid* OR carotenoid* OR glucosinolate* OR organosulfur OR organosulphur OR resveratrol OR quercetin OR curcumin OR fisetin OR anthocyanin* OR sulforaphane OR garlic) AND ("oxidative stress" OR antioxidant* OR inflammation OR cytokine* OR "NF-κB" OR NLRP3 OR SIRT1 OR microbiota OR dysbiosis OR "short-chain fatty acid*" OR SCFA OR butyrate OR "bile acid*" OR glutamate OR GABA)

Table S1B. Data-charting form (fields for extraction)

**ID**	**Database**	**Year**	**Country**	**Model**	**Design**	**Sample (n, sex)**	**Phytonutrient/Class**	**Dose/Form/Duration**	**Primary Mechanism**	**Outcomes**	**DOI/PMID**
**e.g., 001**	PubMed	2025	Egypt	Reserpine-induced rat model of FM	Animal study	60 rats (12 rats/group)	Anthocyanin (polyphenol)	200 mg/kg; 31 day	miR-145-5p, and miR-451a; serotonin; H_2O_2; TNF-alfa, caspase-3	Pain, depression, cognitive impairment	10.1016/j.intimp.2025.114965

Section S1C. Deduplication rules

Import order: PubMed → Scopus → Web of Science → ScienceDirect. Match keys: DOI > Title (lowercased, punctuation-stripped) > Year > First author. Automated matching in Mendeley followed by manual verification for unmatched items.

Section S1D. Controlled exclusion reasons

E1: In vitro models

Cell-only studies that do not include FM-relevant behavioural/clinical endpoints or validated mechanistic readouts with in vivo applicability (e.g., pain behavior, FIQR-related markers, microbiome/SCFA, SIRT1 activity in tissues).

E2: Not primary research

Rationale: Editorial, commentary, letter, narrative review, perspective, protocol without results, preprint duplicate of a published paper.

E3: No FM or FM-relevant mechanism

Rationale: No clinical FM diagnosis (ACR criteria) and no accepted FM-like animal model (e.g., reserpine, intermittent cold stress).

E4: No phytonutrient exposure

Rationale: Pharmacologic drugs or mixed nutraceuticals where the phytonutrient component is absent or not isolable; extract lacks composition/standardization; dose/formulation not reportable.

E5: The outcomes do not match the predefined mechanisms or clinical endpoints

Rationale: No data on redox (MDA/4-HNE, SOD/CAT/GPx, PON-1/NO, TAC/TOS/OSI), inflammation (TNF-α/IL-6/IL-8/IL-10, NLRP3), gut-microbiota/metabolites (SCFAs, bile acids, glutamate/GABA), or SIRT1; and no patient-relevant outcomes (pain, FIQR, fatigue, sleep, GI).

E6: Full text not retrievable

Rationale: The full text cannot be obtained through institutional search, interlibrary loan, or author contact (document attempts).

E6: Duplicate/Overlap

Rationale: Same cohort/model/time-point as another included record; retain the most complete/primary report.
